# Patterns of Post-Endodontic Restoration: A Nationwide Survey of Dentists in Turkey

**DOI:** 10.3390/ijerph19031794

**Published:** 2022-02-04

**Authors:** Sıla Nur Usta, Begüm Cömert-Pak, Eda Karaismailoğlu, Ayhan Eymirli, Derya Deniz-Sungur

**Affiliations:** 1 Department of Endodontics, Faculty of Dentistry, University of Hacettepe, Ankara 06230, Turkey; begumcomert.bmcm@gmail.com (B.C.-P.); ayhaneymirli@gmail.com (A.E.); gusefdeniz@yahoo.com (D.D.-S.); 2 Department of Medical Informatics, Gulhane Faculty of Medicine, University of Health Sciences, Ankara 06018, Turkey; edaozturk82@gmail.com

**Keywords:** composite resins, endodontics, post core technic

## Abstract

Developments in materials and techniques, geographical locations, age, specialty, and affiliations of dental practitioners affect the preference of post-endodontic restoration. Thus, this survey aimed to evaluate the trends of dentists in Turkey in terms of post-endodontic restorations. An anonymous survey containing 10 questions regarding demographics, post-endodontic restoration patterns, and factors affecting restoration selection was electronically delivered to the dentists registered in the database of the Turkish Dental Association. The data were analyzed using by a chi-square test and ordinal logistic regression analysis. A total of 1093 surveys from 20,564 participants were collected with a response rate of 5.3%. Half of the participants (52%) preferred composite resins for post-endodontic restorations. Usage of posts was less prevalent amongst prosthodontists and dentists with clinical experience of more than 20 years compared to endodontists (*p* < 0.001) and dentists with clinical experience of less than 5 years (*p* = 0.004). More than half of the participants (56%) utilized fiber posts. Composite resins and fiber posts were the most common preferences in post-endodontic restoration. Endodontists had a higher tendency to use posts than prosthodontists and general dental practitioners.

## 1. Introduction

Endodontically treated teeth (ETT) are prone to root fractures due to substance loss [1]. Therefore, the placement of proper coronal restoration after endodontic treatment is an essential parameter for the tooth’s survival. A suitable restoration not only restores the tooth aesthetics and function but also prevents microbial leakage [2,3]. However, no particular causal relationship between fractures and the type of restoration has been established. Studies have tried to find which material or technique is suitable for ETT rehabilitation [4].

Different restoration patterns have been used after endodontic treatments, such as partial- or full-coverage crowns [5], direct resin composites or amalgam fillings [6], and posts and cores [7]. Additionally, the availability of adhesive techniques has expanded the restoration options for the clinician [8]. Therefore, contemporary approaches such as endocrowns [9], computer-assisted designing and computer-assisted milling (CAD/CAM) posts [10], and fiber-reinforced composites [11] related to the advances in technology have also been preferred since their elastic modulus is closer to that of dentin, and they have a better aesthetic outcome.

Post placement is generally suggested if the residual tooth structure is insufficient to support a core [3]. A large variety of post designs and materials has been introduced to increase the resistance of the remaining dental tissue of ETT [12,13,14]. Cast posts and cores [3] and prefabricated metal posts have been widely used [15]. However, fiber posts have gained popularity due to their flexibility and modulus of elasticity, which is more similar to that of dentin when compared to metal posts [16].

It has been reported that the primary cause of endodontic treatment failure is due to restoration failure rather than a failure of the endodontic treatment itself [17]. Therefore, a proper post-endodontic restoration is a crucial factor for the success of endodontic therapy. Factors such as the economic and aesthetic outcome, periodontal condition of the tooth, the remaining tooth structure, tooth location, and the habits and expectations of the patient should be considered in the treatment plan.

The selection of post-endodontic restoration is affected by several factors, such as developments in materials and techniques, geographical location, age, specialty, and affiliations of dental practitioners [18]. Moreover, inconsistencies among newly developed dental materials, scientific literature, what is taught in dental school, and what is actually applied in clinics have been reported [19]. Therefore, survey-based studies are beneficial tools that evaluate the applicability and the factors affecting the choices of contemporary restoration alternatives. Although several surveys have been carried out in various countries [20,21,22,23], no study has revealed the trends of post-endodontic restorations carried out by dentists in Turkey. Thus, this study aimed to evaluate the different trends in post-endodontic restoration preferences amongst Turkish dentists via survey.

## 2. Materials and Methods

The present questionnaire was validated using face validity by experts with experience and people who understand the topic. They evaluated whether the questions effectively captured the topic under investigation. Secondly, a statistician checked the survey for common errors such as double-barreled, confusing, and leading questions. The Ethical Board and Commission of Hacettepe University also approved this anonymous survey. Potential participants of the survey were all the dental practitioners registered in the database of the Turkish Dental Association. All of them were contacted electronically through an official email from the Turkish Dental Association. An explanatory letter containing the instructions, the name of the authors, and the purpose of this study was also included. A modified version of the questionnaire used by Morgano et al. [18] was re-designed by two researchers (SNU and DDS) who have experience in post-endodontic restorations, reducing the number of questions to 10 and updating some of them to account for the new materials introduced in recent years. The first four questions were related to basic demographic details. The last six were about the patterns of post-endodontic restoration, materials and methods, and factors affecting the choices of contemporary restoration alternatives. Supplementary Material Table S1 shows all 10 questions.

The survey was available through an online survey system between the 15 July 2020 and the 15 October 2020. The survey was conducted anonymously, so those who did not respond to the survey could not be identified, and only anonymized data from the respondents were included in the study.

### Statistical Analysis

The data were statistically analyzed using SPSS for Windows Version 21.0 (IBM Inc., Chicago, IL, USA). Categorical variables were summarized as frequency (percentage) and were compared by a chi-square test. The post hoc analysis was implemented to explore differences between groups. Demographics such as clinical experience, affiliation, specialty, and geographic region were selected as variables for the frequency of using posts for the restoration of ETT. The associations mentioned above were evaluated by using ordinal logistic regression analysis. A *p*-value of 0.05 or less was considered statistically significant.

## 3. Results

The survey was electronically delivered to 20,564 dental practitioners registered in the database of the Turkish Dental Association. A total of 1093 surveys were completed, and the response rate was determined as 5.3%.

### 3.1. Demographic Information

**Figure 1:**
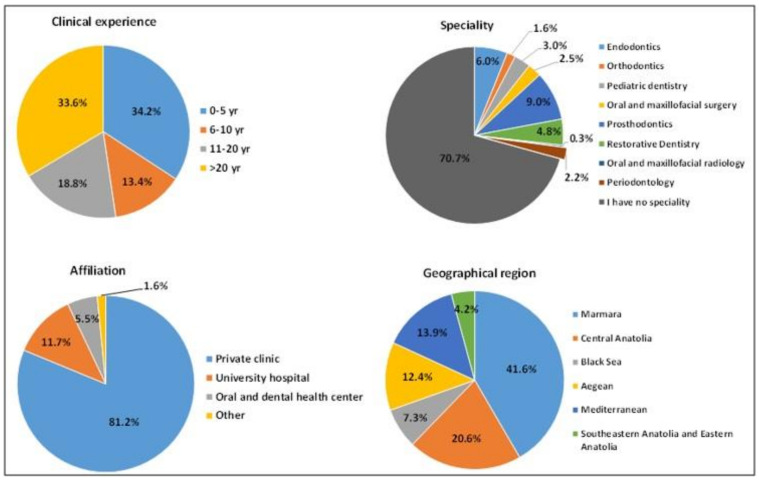
Demographics of the participants.

The demographics of the dental practitioners are shown in Figure 1. Most of the participants had clinical experience of more than 20 years (34%) and less than 5 years (34%). Most of the participants were general dental practitioners without a specialty (71%), while only 6% were endodontists, and 9% were prosthodontists. The remaining 10% were other specialists such as oral surgeons, orthodontists, pedodontists, periodontologists, and oral and maxillofacial radiologists. Moreover, most practitioners practiced in private clinics (81%). Furthermore, most of the respondents were from the Marmara (42%) and the Central Anatolia regions of Turkey (21%).

### 3.2. Patterns in Post-Endodontic Restorations

**Figure 2:**
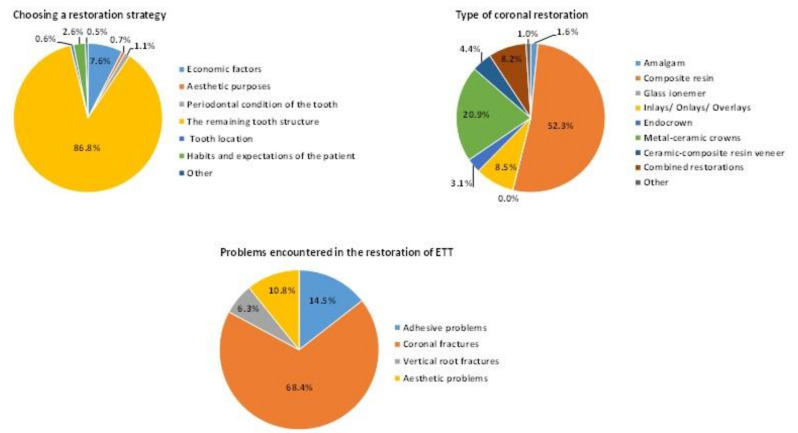
Patterns in post-endodontic restorations.

Results related to patterns in post-endodontic restorations are depicted in Figure 2. According to the results, the remaining tooth structure (87%) was the most influential factor in the post-endodontic restoration strategy. Half of the participants (52%) and all specialties except prosthodontists mostly preferred composite resins for post-endodontic restorations. Prosthodontists had a significantly higher tendency to use metal–ceramic crowns compared to composite resins (*p* < 0.05). Moreover, those with less than 5 years or more than 20 years of clinical experience were also prone to using composite resins. Additionally, composite resins were commonly used than metal–ceramic crowns in university hospital settings (*p* < 0.05). Composite resin was also the most preferred material in all geographic regions.

**Figure 3:**
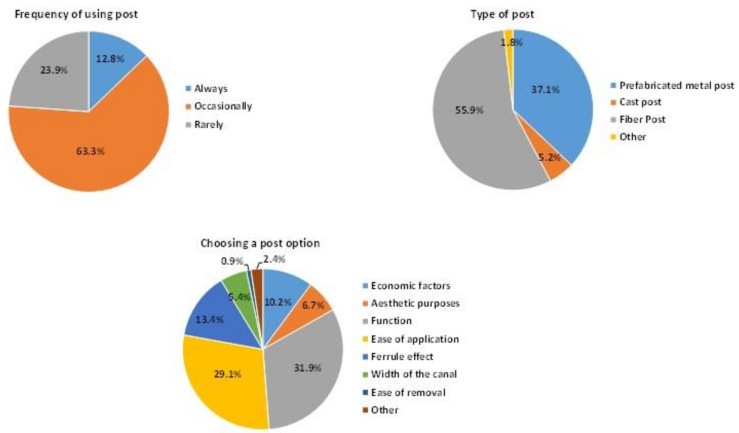
The results associated with using posts.

The results on the usage of posts are shown in Figure 3. Sixty-three percent of the participants used posts occasionally in post-endodontic restoration. According to the responders, function (32%) and ease of application (29%) were the most influential factors in choosing a post. More than half of the participants (56%) utilized fiber posts. Fiber posts were more prevalent among participants with less than 5 years or more than 20 years of clinical experience (*p* < 0.05). Additionally, although fiber posts were mostly the preferred material in private clinics and university hospitals, prefabricated metal posts were used more often in oral and dental health centers than fiber posts (*p* < 0.05).

A coronal fracture was the most common problem (69%) associated with post-endodontic restorations, followed by adhesive and aesthetic problems. In addition to this, a statistically significant difference was found between coronal fractures and adhesive problems for participants with less than 5 years or more than 20 years of clinical experience (*p* < 0.05).

### 3.3. Effects of Demographic Factors on Using Posts

The effects of the demographics of the responders, which were determined as clinical experience, affiliation, specialty, and geographic region, on the frequency of using posts in the post-endodontic restoration were analyzed with ordinal logistic regression analysis. The results are presented in Table 1. Usage of posts was less frequent amongst prosthodontists (*p* < 0.001), periodontists (*p* = 0.001), and general dental practitioners (*p* = 0.004) compared to endodontists. It was found that participants with clinical experience of more than 20 years used posts 1.6 times less often than those with clinical experience of less than 5 years (*p* = 0.004; OR = 0.633; CI = 0.465–0.861). Moreover, posts were used 1.7 times less often in university hospitals than private clinics (*p* = 0.043; OR = 0.591; CI = 0.355–0.983). Furthermore, usage of posts was more common among participants in the Aegean region compared to Marmara (*p* = 0.024; OR = 1.585; CI = 1.062–2.366).

## 4. Discussion

The present survey study aimed to evaluate the different restoration patterns of dentists in Turkey and to update the newly developed restoration strategies and materials used in post-endodontic restorations. This study has a retrospective character, which allowed the respondents to give subjective estimates to the questions, especially the quantitative ones. Therefore, these answers may deviate from adequate clinical numbers. Furthermore, it must be noted that the results are entirely based on answers from dentists with an interest in this survey; thus, caution must be exercised in generalizing these results among all Turkish dentists.

According to the responses, composite resins (52%) were the most preferred post-endodontic restoration type, followed by metal–ceramic crowns (21%) and inlays/onlays/overlays (9%). Although post-and-core restorations were considered essential in teeth with endodontic treatment due to the extensive loss of tooth structure [24], restorations without post-and-core build-up have gained popularity due to their minimal invasiveness, increased adhesive properties, and less intensive clinical procedure [25]. It has been reported that resin composite restorations showed an excellent success rate in teeth with adequate remaining tooth structure [26]. Furthermore, digital systems such as CAD/CAM have become a well-accepted technology for various applications, including post-endodontic restorations. They exhibit increased mechanical strength, prevent porosity within the restorations, and reduce the chair-side time [27].

The remaining tooth structure (87%) was the most influential factor in choosing the restoration patterns, and coronal fractures (68%) were the most common problem encountered in post-endodontic restorations. It has been reported that the restorative design of cusp coverage could provide better protection for the remaining teeth by redistributing the stress [28]. However, the literature has a significant number of contradictory reviews regarding this issue. At the same time, some clinicians maintain that post-endodontic restorations should be performed by full-cusp coverage, while others claim that no ETT require full-cusp coverage protection. Therefore, it should be considered only when the caries’ destruction and tooth structure loss have been extensive [29].

The presented data show that 63% of the participants reported utilizing a post occasionally, while 24% utilized them rarely, and 13% always utilized them. This finding is similar to those of other surveys published in the literature [17,18,19,20,21,22,23,24,25,26,27,28,29,30,31]. Previously, it was mentioned that all teeth with root canal treatment should be restored with a post [32]. However, later studies indicated that posts should only be used in cases of extensive loss of coronal structure, and posts may decrease fracture resistance when a proper ferrule is not established [33,34]. Since the coronal structure of the tooth can become vulnerable after endodontic treatments, usage of the post may vary according to the case selection.

According to the results of the survey, the function (32%) of posts was the most influential factor in choosing a post type. Posts provide sufficient retention for the core, distributing the functional stress to the root [35]. Hence, a proper post type keeps the tooth structure functional and reduces the risk of vertical fractures. It also improves the biomechanical behavior, survival rate, and durability of teeth that have undergone root canal treatment [36,37]. Notable success and survival rates of ETT with post restorations were observed in a private practice setting [38]. The ease of application was significantly the most influential factor in choosing a post type for general dental practitioners (*p* < 0.05). Interestingly, this was not reported as an influential factor by prosthodontists and specialists in restorative dentistry. This result might be explained by specialty training, which provides more advanced techniques and knowledge in guidelines to specialists compared to general dental practitioners.

Fiber posts (56%) were widely preferred for post-endodontic restorations, while prefabricated metal posts (37%) and cast posts and cores (5%) were less preferred. This result is in agreement with the other studies in the literature [20,21,22]. Usage of prefabricated metal posts and cast posts and cores has been decreasing due to several disadvantages in their procedure, such as biological problems owing to microleakage, insufficient aesthetic properties, corrosion, and increased fracture risk related to the non-homogeneous distribution of stress [39,40,41]. The rising popularity of fiber posts may be due to their superior aesthetic properties and the time efficiency of the procedure [37]. Besides, they also reduce the risk of vertical root fracture since they have a modulus of elasticity similar to that of dentine [42]. Although clinical studies show only minor differences between different posts [43], the placement costs for different posts may differ in laboratory costs or luting efforts [44]. These results have not been reported in other countries such as the UK and Sweden, where cast posts and prefabricated metal posts were reported to be preferred [31,32,33,34,35,36,37,38,39,40,41,42,43,44,45]. However, these studies were performed during 1995–2001, before the fiber post had gained the popularity it experiences today. Therefore, the period of these studies may affect their result; therefore, they should be further discussed and updated.

The design of this survey study may be assumed as a limitation as it relies on the dental practitioner’s individual reports, which provide a low level of evidence, especially when the low response rate is considered (5.3%). Since the survey was anonymous and delivered to many dentists, those who did not respond to the survey could not be identified. Moreover, since the survey was delivered via email to the dentists registered in the database of the Turkish Dental Association, some of them might not have been received tit because of spam emails or inactive usage. Another limitation of this survey is that restoration strategies for anterior and posterior teeth were not investigated separately. Therefore, this survey is still unclear about post-endodontic restoration patterns regarding the tooth type. The present survey reflects only the general opinions of the participants about post-endodontic restorations. Further studies are required to confirm these findings with a greater number of questions and a larger sample size.

## 5. Conclusions

Within the limitations of the present survey, it showed that dentists in Turkey use the current post-endodontic restoration alternatives depending on several clinician-related factors, such as the years of clinical experience, specialization, work setting, and geographic region. Endodontists had a higher tendency to use posts than prosthodontists and general dental practitioners. Composite resins and fiber posts were the most common preference in post-endodontic restoration. Since the results do not represent all Turkish dentists, it is difficult to derive a generalizable, clear, and structured restoration concept.

## Figures and Tables

**Table 1:** Multivariate ordinal logistic regression model on the association of the usage of posts with several demographic factors amongst the participants of the survey.

Variables		*p*-Value	OR	95% CI of OR	
How long have you been practicing dentistry?	0–5 years	Reference			
	6–10 years	0.659	1.094	0.733	1.635
	11–20 years	0.476	1.141	0.794	1.638
	>20 years	0.004 **	0.633	0.465	0.861
What is your specialty?	Endodontics	Reference			
	Orthodontics	0.693	0.799	0.262	2.435
	Pediatric Dentistry	0.198	0.574	0.247	1.335
	Oral and Maxillofacial Surgery	0.519	0.748	0.309	1.809
	Prosthodontics	<0.001 ***	0.180	0.093	0.349
	Restorative Dentistry	0.967	0.984	0.454	2.130
	Oral and Maxillofacial Radiology	0.060	0.106	0.010	1.096
	Periodontology	0.001 **	0.197	0.075	0.521
	General Dental Practitioners	0.004 **	0.450	0.263	0.770
What kind of institution do you practice in?	Private Clinic	Reference			
	University Hospital	0.043 *	0.591	0.355	0.983
	Oral and Dental Health Center	0.149	1.477	0.869	2.510
	Other	0.385	1.521	0.590	3.922
Which geographic region do you practice in?	Marmara Region	Reference			
	Central Anatolia Region	0.550	1.110	0.788	1.564
	Black sea Region	0.394	1.241	0.756	2.036
	Aegean Region	0.024 *	1.585	1.062	2.366
	Mediterranean Region	0.142	1.333	0.908	1.958
	South-eastern Anatolia and Eastern Anatolia Regions	0.844	1.066	0.565	2.010

Abbreviations: OR: odds ratio; CI: confidence interval; * *p* < 0.05, ** *p* < 0.01, *** *p* < 0.001.

## Data Availability

All data are contained within the article results. Further information may be provided upon request to the corresponding author.

## Supplementary Materials

The following supporting information can be downloaded at: https://www.mdpi.com/article/10.3390/ijerph19031794/s1. Table S1: The 10 questions about the basic demographic details of the respondents, patterns of post-endodontic restoration, materials and methods, and factors affecting the choices of contemporary restoration alternatives.
