# Supplementary material for: Patterns of Post-Endodontic Restoration: A Nationwide Survey of Dentists in Turkey
Source: Int J Environ Res Public Health. 2022 Feb 4;19(3):1794. doi: 10.3390/ijerph19031794 (PMC8834666; doi:10.3390/ijerph19031794)
Supplement: Supplementary file 1 [file ijerph-19-01794-s001.zip › ijerph-1542784-supplementary.pdf]

Table S1. The 10 questions about the basic demographic details of the respondents, patterns of post-endodontic restoration, materials, and methods, and factors affecting the choices of contemporary restoration alternatives.

QUESTIONS	ANSWERS
**Q1:** How long have you been practicing dentistry?	0-5 years<br>6-10 years<br>11-20 years<br>>20 years
**Q2:** What is your specialty?	Endodontics<br>Orthodontics<br>Pediatric dentistry<br>Oral and maxillofacial surgery<br>Prosthodontics<br>Restorative dentistry<br>Oral and maxillofacial radiology<br>Periodontology<br>General dental practitioners without any special training
**Q3:** Which institution do you practice in?	The private clinic<br>University hospital<br>Oral and dental health center<br>Other
**Q4:** Which geographic region do you practice in?	Dental practitioners were able to write which city they work in this section.
**Q5:** What is the most influential factor in choosing your post-endodontic restoration strategy?	Economic factors<br>Aesthetic purposes<br>Periodontal condition of the tooth<br>The remaining tooth structure<br>Tooth location<br>Habits and expectations of the patient<br>Other
**Q6:** Which coronal restoration type do you prefer mostly in post-endodontic restoration?	Amalgam<br>Composite resin<br>Glass ionomer<br>Inlays/ Onlays/ Overlays<br>Endocrown<br>Metal-ceramic crowns<br>Ceramic-composite resin veneers<br>Combined restorations<br>Other
**Q7:** How frequently do you use a post for post-endodontic restoration?	Always<br>Occasionally<br>Rarely
**Q8:** What is the most influential factor in choosing your post option?	Economic factors<br>Aesthetic purposes<br>Function<br>Ease of application<br>Ferrule effect<br>The width of the canal<br>Ease of removal when a problem has occurred<br>Other

**Q9:** Which type of post do you use most frequently for post- endodontic restoration?	Prefabricated metal posts<br>Cast posts-and-cores<br>Fiber posts<br>Other
**Q10:** What is the most common problem you encounter in post-endodontic restorations?	Adhesive problems<br>Coronal fractures<br>Vertical root fractures<br>Aesthetic problems
